# Effects of impulsivity and emotions on time perception: Laboratory behavioral measures

**DOI:** 10.1177/03010066251316457

**Published:** 2025-02-10

**Authors:** Diana Moreira, Andreia Azeredo, Ângela Leite, Fernando Barbosa

**Affiliations:** Universidade Católica Portuguesa, Portugal; University of Porto, Portugal; Institute of Psychology and Neuropsychology of Porto – IPNP Health, Portugal; Centro de Solidariedade de Braga/Projecto Homem, Portugal; Observatory Permanent Violence and Crime (OPVC), FP-I3ID, Portugal; Universidade Católica Portuguesa, Portugal; University of Porto, Portugal; Universidade Católica Portuguesa, Portugal; University of Porto, Portugal

**Keywords:** impulsivity, BIS/BAS scales, time perception, time estimation, emotions

## Abstract

Impulsivity is consistently linked to various problematic behaviors, including aggression, substance abuse, pathological gambling, risky driving, and numerous psychopathological disorders such as attention deficit hyperactivity disorder and personality disorders. This study aims to investigate the relationship between self-reported impulsivity, measured by the Behavioral Inhibition/Behavioral Activation Scales, and emotional states (pleasant, unpleasant, or neutral), in the context of time estimation deviations. A time estimation task was administered to 129 adult participants (88 females) from the community to assess this deviation. The findings reveal that participants underestimated time across all emotional conditions, enhancing our understanding of how impulsivity relates to time perception. Therefore, it is crucial to continue neuropsychophysiological research on impulsivity to explore its causes, manifestations, and connections with other aspects of cognitive and affective functioning. This research will lead to a more precise definition and comprehensive understanding of impulsive behavior.

Impulsive behavior is shaped by a complex set of processes, often characterized by limited action planning due to the potential for negative consequences (Moreira et al., 2016). Traditionally, impulsivity was perceived negatively, associated primarily with dysfunctional outcomes. It is frequently linked to problematic behaviors such as aggression (Vigil-Colet & Codorniu-Raga, 2004), substance abuse (Colder & Chassin, 1997), pathological gambling (Blaszczynski et al., 1997), risky driving (Paaver et al., 2006), and various psychopathological disorders, including attention deficit hyperactivity disorder (ADHD) and personality disorders. However, impulsivity can also lead to positive and functional outcomes (Dickman, 1990). Dickman's (1990) concept of functional impulsiveness aligns with the Reinforcement Sensitivity Theory developed by Gray and McNaughton (2000), suggesting that impulsivity can have adaptive components depending on the context.

Research methods for studying impulsivity typically fall into two primary categories: laboratory-based behavioral tasks and self-report measures. Laboratory tasks offer objective insights into impulsivity through controlled experiments. These include response inhibition tasks, which evaluate the ability to suppress impulsive reactions; reward delay tasks, which measure the preference for immediate versus delayed rewards; and behavioral timing tasks, which assess the perception and estimation of time intervals (Dougherty et al., 2005; Evenden, 1999; Pleskac et al., 2019; Zhu et al., 2017).

Self-report measures encompass various questionnaires designed to assess impulsivity traits. Notable among these are the Temperament and Character Inventory (Cloninger, 1999), the Dickman Impulsivity Inventory (Dickman, 1990), the Impulsiveness Questionnaire (Eysenck et al., 1985), the Barratt Impulsiveness Scale (BIS-11, Patton et al., 1995), and the Sensation Seeking Scale (Zuckerman, 1994). Each of these questionnaires focuses on different aspects of impulsivity, leading to variations in assessment outcomes (Havik et al., 2012).Carver and White (1994) developed the BIS/BAS scales based on Gray's motivational systems theory. Gray, expanding on Fowles’ work (1980), suggested that individual personality traits are influenced by the sensitivity and reactivity of two core motivational brain systems: the Behavioral Inhibition System (BIS) and the Behavioral Activation System (BAS). These systems are used to explain differences in various forms of psychopathology, including psychopathy (Fowles, 1980), bipolar disorder (Depue & Iacono, 1989), anxiety disorders (Gray & McNaughton, 2000), and depression (Harmon-Jones & Allen, 1997). The BIS/BAS scales are brief self-report measures assessing the reactivity of these systems. Initially, the scales included one subscale for BIS and three for BAS: Reward Responsiveness (RR), Drive (D), and Fun Seeking (FS). The BAS subscales focus on different aspects of reward processing: RR assesses emotional responses to rewards, D measures persistence in pursuing goals, and FS evaluates the desire for new rewards and the tendency to impulsively approach them (Moreira et al., 2015). The BIS items measure distress associated with negative events, including concerns, anxieties, and fears. The division of the BAS into three subscales highlights the ongoing debate about how BAS sensitivity manifests (Carver & White, 1994). Some researchers argue that functional impulsivity (reward responsiveness) and dysfunctional impulsivity (the traditional view of impulsivity) are distinct characteristics (Franken & Muris, 2006; Smillie & Jackson, 2006).

Research findings indicate that individuals with faster cognitive processing speeds—often described as having a quicker internal clock or pacemaker—tend to display higher levels of activity (Barratt, 1983). Additional studies propose that impulsive individuals might perceive time differently, potentially contributing to their challenges with delaying gratification. A systematic review by Moreira et al. (2016) revealed that patients with personality disorders (Bauer, 2001; Berlin et al., 2005, 2010; Berlin & Rolls, 2004; Havik et al., 2012; Schulreich et al., 2013), stimulant addiction (Wittmann et al., 2007), heroin addiction (Petry et al., 1998), cocaine abuse (Wittmann et al., 2007), alcoholism (Klingemann, 2001; Smart, 1968), pathological gambling (Hodgins & Engel, 2002; Zauberman et al., 2009), and traumatic brain injuries, including orbitofrontal (Berlin et al., 2005; Minkwitz et al., 2012), and parietal lesions (Alexander et al., 2005; Bueti et al., 2008; Petrovici & Scheider, 1994), generally overestimate the passage of time. This tendency is often associated with high levels of impulsivity (Moreira et al., 2016).

Other experimental findings reveal a more complex relationship between impulsivity and time perception (TP). For instance, children and adults with ADHD exhibit difficulties in processing time intervals, ranging from milliseconds to several seconds, across various sensorimotor timing tasks (Barkley et al., 2001; Smith et al., 2002). Although these individuals are more likely to devalue delayed rewards, their verbal time estimates for durations of several seconds align closely with those of individuals without ADHD (Burle & Casini, 2001; Kahneman & Tversky, 1979). Interestingly, individuals without ADHD also demonstrate a tendency to devalue delayed rewards. Additionally, smokers experiencing intense cravings report that time seems to pass more slowly (Sayette et al., 2005). This perception could be partly attributed to the heightened arousal from the craving for tobacco, or it might be due to an increased awareness of time while waiting for an opportunity to smoke (Sewell et al., 2013).

However, few studies have explored the intricate relationships between affective states, impulsivity, and TP. Research has shown that individuals prone to boredom, those suffering from depression, and oncology patients with high levels of anxiety often perceive time as dragging and overestimate durations in time estimation tasks (Somov, 2000). In such situations, acute stress can shift attention away from ongoing thoughts and actions to the passage of time, leading to an overestimation of its duration. Similarly, impulsive individuals frequently experience distress when their typical impulsive behaviors are restricted, such as during waiting periods, which results in a heightened focus on the passage of time and subsequent overestimation (Somov, 2000). Additionally, it is possible that more impulsive individuals may have faster internal clocks, combined with increased central nervous system activation, which could contribute to their tendency to overestimate time.

The impact of emotions on TP appears to depend on the emotional valence of the stimuli. During time estimation tasks, participants typically overestimate the duration of unpleasant images (often categorized as negative emotions, include a wide range of feelings that people generally find distressing or difficult to experience) more than pleasant ones, even when the activation levels of both types of images are controlled (Gil & Droit-Volet, 2009, 2011a, 2011b; Tipples, 2008, 2011).

Unpleasant emotions, often categorized as negative emotions, include a wide range of feelings that people generally find distressing or difficult to experience. Some common unpleasant emotional states include anxiety (feeling of worry, nervousness, or unease, typically about an imminent event or something with an uncertain outcome; Freud et al. (1977) discussed anxiety as a psychological state that arises from repressed feelings and desires; Sartre et al. (2022) views anxiety as a fundamental part of the human condition, especially in confronting freedom and choice; fear (distressing emotion induced by perceived danger or threat; Darwin, 1993, discusses fear as a primal emotion with physiological responses and LeDoux, 2012, explored the brain's role in fear, particularly the amygdala's function in processing fear); sadness (feeling of sorrow or unhappiness, often in response to loss, disappointment, or helplessness; Kubler-Ross, 1969, on her work on the five stages of grief includes sadness as a key emotional stage when people confront death or significant loss); guilt (feeling of responsibility or remorse for some offense or wrongdoing; Freud, 1989, (Superego) theorized that guilt arises from the conflict between the ego and the superego (conscience), when moral values are violated); shame (painful feeling of humiliation or distress caused by consciousness of wrong or foolish behavior; Miceli & Castelfranchi, 2018, emphasized the difference between shame and guilt); and anger (strong feeling of annoyance, displeasure, or hostility); Kassinove (1995) studied anger as a mood state of everyday life.

The underlying mechanisms of attention or activation-induced changes in TP among impulsive individuals may complement each other, contributing to altered temporal perception (Burle & Casini, 2001). Some researchers suggest that exposure to emotional facial expressions is perceived as lasting longer compared to neutral expressions because emotions provoke a heightened state of activation, which may accelerate the internal pacemaker (Dirnberger et al., 2012; Droit-Volet et al., 2004). While these effects might be more pronounced in individuals with higher levels of impulsivity, current research does not provide conclusive evidence to support this hypothesis. In fact, some studies reported that people tend to overestimate the duration of “unpleasant” images (Bradley et al., 2001; Droit-Volet et al., 2004), but not in individuals with higher level of impulsivity. Regardless of the findings synthesized above, some studies did not report TP alterations in the same type of groups or conditions. For example, according to Berlin et al. (2010) individuals with borderline personality disorder (BPD) seem to have preserved TP, which is inconsistent with evidences of time production deficits from other studies of BPD patients (e.g., Berlin & Rolls, 2004). Also, despite evidences of temporal distortions in addicted individuals (e.g., Hodgins & Engel, 2002), it is not clear if psychological distress plays a main role in this effect, instead of addiction per se (MacKillop et al., 2006).

This study aimed to explore the relationship between self-reported impulsivity, assessed using the BIS/BAS scales—focusing on the BIS, BAS-D, and BAS-FS subscales—and emotional contexts (pleasant, unpleasant, or neutral) on deviations in time estimation. We hypothesized that (a) there is a positive correlation between higher impulsivity and a greater tendency to overestimate time intervals, as indicated by higher scores on the BAS-FS and BAS-D subscales and lower scores on the BIS subscale. Furthermore, we proposed that (b) higher scores on the BIS subscale would be associated with the underestimation of time intervals, while (c) higher scores on the BAS-FS and BAS-D subscales would be linked to the overestimation of time intervals. Additionally, we expected to observe (d) a greater overestimation of time in the unpleasant emotional condition compared to the pleasant and neutral conditions.

## Method

### Participants

Prior to participation, participants were informed about the objectives of the study, and written informed consent was obtained from each of them. Initially, the sample consisted of 136 participants. Six were excluded for scoring below the cutoff point suggested for the Portuguese population on the Montreal Cognitive Assessment (MoCA; Simões et al., 2008), and one participant was excluded for obtaining z > |3| θ values in at least one of the experimental conditions. No other participants were excluded based on additional self-reported exclusion criteria, such as alcohol or substance abuse, medication use, psychopathologies or neuropathologies, or sensorimotor deficits that might affect task performance. The study received approval from the local ethics committee, and all participants volunteered anonymously without financial compensation.

Thus, this study comprised 129 adult participants (88 women) from the northern region of Portugal, with an average age of 29.6 years (*SD *= 8.50) and an average of 14.8 years of schooling (*SD *= 4.01). Participants were recruited through public announcements, social networks, and referrals using the snowball technique.

### Materials

#### BIS/BAS (Carver & White, 1994; Portuguese version by Moreira et al., 2015)

This instrument was utilized to assess impulsivity by examining behavioral indicators of activation in the BAS and BIS. The Portuguese version of the BIS/BAS scales consists of 16 statements, rated on a 4-point Likert scale, ranging from 1 (*very true for me*) to 4 (*very false for me*), indicating agreement level. Cronbach's α values for the scales (*N *= 916; 438 women) BIS (two items; min = 2, max = 8), BAS-RR (eight items; min = 8, max = 32), BAS D (four items; min = 4, max = 16), and BAS-FS (two items; min = 2, max = 8) were .77, .79, .81 and .64, respectively (Moreira et al., 2015). The analysis revealed four factors with eigenvalues exceeding 1, collectively explaining 56.8% of the total variance. In this study, Cronbach's α values for the BIS, BAS-RR, BAS-D, and BAS-FS scales were .60, .62, .75, and .63, respectively. As all α values were ≥ .60, indicating satisfactory internal consistency (Field, 2009).

In this study, impulsivity was measured using BIS scores, along with BAS-FS and BAS-D, consistent with previous research methodologies (Franken et al., 2005; Knyazev et al., 2004; Poythress et al., 2008).

#### Stimuli

The stimuli consisted of 45 color images extracted from the *Nencki Affective Picture System*^1^ (NAPS; Marchewka et al., 2014). The images in this database are preevaluated for valence using a 9-point scale, where values closer to 1 represent *negative valence*, values closer to 9 represent *positive valence*, and values around 5 represent *neutrality*.

The stimulus set comprised an equal number of images in three categories: (a) positive valence images (*M *= 7.94, *SD *= 0.20), representing the pleasant stimuli condition; (b) negative valence images (*M *= 1.94, *SD *= 0.25), representing the unpleasant stimuli condition; and (c) neutral images (valence: *M *= 5.07, *SD *= 0.14), representing the neutral stimuli condition. These valence ratings were derived from the NAPS image database (Marchewka et al., 2014).

The stimuli were displayed at the center of a 17″ computer monitor, with participants seated approximately 80 cm away from the screen. Task administration and response collection were facilitated using E-Prime 2.0 software (2011, Psychology Software Tools, Inc., Sharpsburg, PA, USA), which controlled the presentation of stimuli and recorded participant responses.

### Procedures

**Figure 1.**
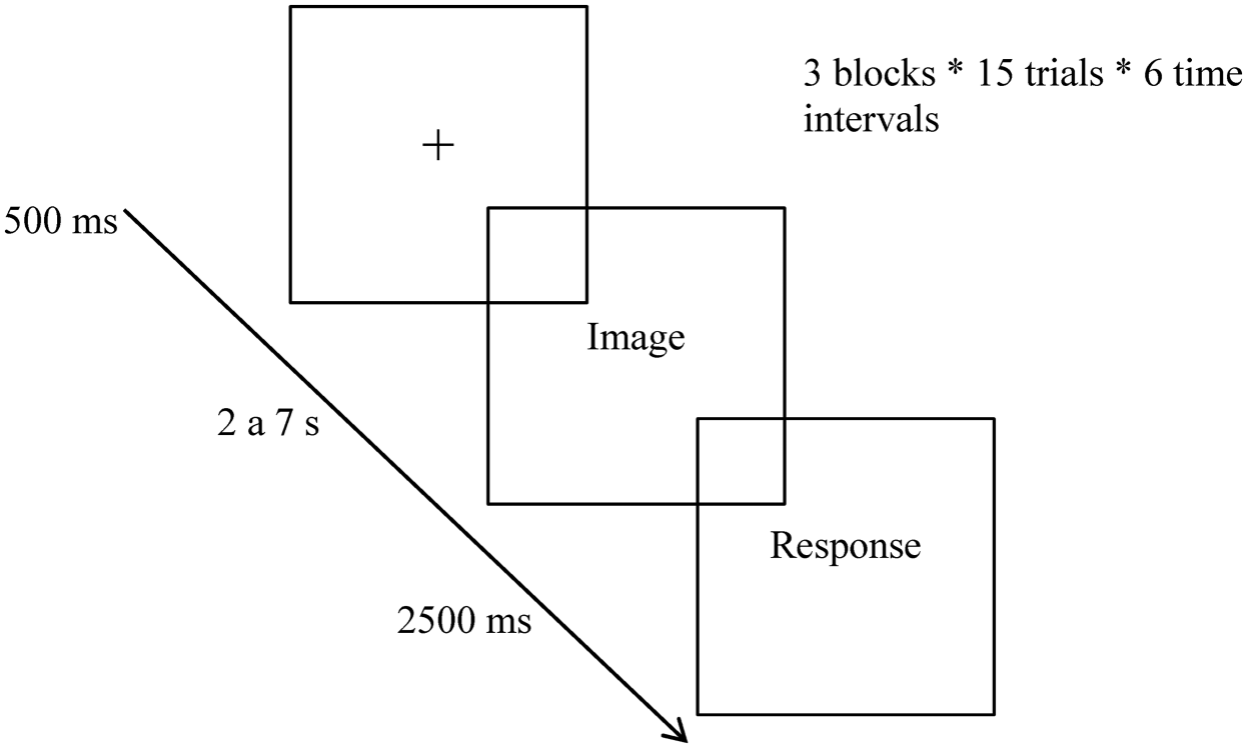
Example of a time estimation trial with emotional interference.

Experimental tasks were programed considering the results of a previous systematic review (Moreira et al., 2016), namely, the time intervals between stimuli varied, according to the studies analyzed, from min = 400 ms to max = 90 s. The time intervals of the experimental tasks to which this work refers ranged from six conditions—from 2 to 7 s. The protocol had an average duration of 40 min for administering screening instruments (MoCA) and assessing impulsivity (BIS/BAS), in addition to 34 min for completing the experimental temporal estimation task. During each trial, participants assessed the duration of exposure to images with varying emotional valences, organized into three blocks: pleasant, unpleasant, and neutral. Pleasant emotions refer to positive affective states that are typically associated with feelings of happiness, joy, satisfaction, contentment, or amusement. These emotions are usually contrasted with unpleasant or negative emotions, such as sadness, anger, fear, or disgust. Neutral emotions refer to affective states that are neither distinctly positive nor negative (absence of valence), including lack of arousal and stability. These blocks were presented consecutively without interruption, and their order was balanced to mitigate order effects and proactive interference (carry-over effect). Within each block, trial durations ranged across six discrete values (ranging from 2 to 7 s), with the order of these intervals pseudo-randomized to prevent consecutive trials with identical durations. Each time interval was presented in 15 trials, resulting in a total of 90 trials per block (15 images × 6 intervals). Participants were informed that the exposure could be from 1 to 9 s and responded between these alternatives. A 2.5-s response window was provided, followed by a 500 ms interstimulus interval to minimize expectancy effects and proactive interference between trials. Participants were instructed to refrain from counting aloud or using any body movements to aid in time estimation (Figure 1). Additionally, participants were instructed not to count aloud or engage in any bodily movements to aid in temporal estimation.

Data collection occurred within a single session at the neuropsychophysiology laboratory, where lighting, temperature, and noise levels were carefully controlled. Participants underwent familiarization with the equipment through six training trials. The blocks of images representing the three emotional conditions were administered in a balanced manner to mitigate order effects, with no pauses between blocks.

### Data Analysis

In the initial phase of data processing and analysis, measures of central tendency (mean, M) and dispersion (standard deviation, SD) were computed. For hypothesis testing, repeated measures analysis of covariance (RM ANCOVA) was conducted using Statistical Packages for Social Sciences (SPSS, version 26, IBM Corp., Armonk, NY). The emotional condition (pleasant, unpleasant, neutral) served as an intrasubject variable, while BIS, BAS-D, and BAS-FS scores (impulsivity measures) were included as covariates to examine their respective effects on temporal estimation (dependent variable), quantified using θ (estimated time/actual time).

Partial eta squared (η²) was calculated as an effect size measure (Cohen, 1992), and the Holm-Bonferroni post hoc test was selected for multiple comparisons due to its robustness compared to the Bonferroni test (Holm, 1979).

Normality assumption was assessed using the Shapiro–Wilk test, supplemented by analysis of skewness (Sk) and kurtosis (Ku) coefficients when violated. As the absolute values of these coefficients typically range between 2 and 7 (Kim, 2013), parametric tests were employed. The assumption of sphericity was evaluated using the Mauchly test, and in case of violation, the Greenhouse–Geisser correction was applied, with the epsilon (ε) value reported.

## Results

The RM ANCOVA, in which the emotional condition (pleasant, unpleasant, neutral) was entered as an intrasubject factor and the scores of the BIS (*M *= 4.016, *SD *= 1.311), BAS-D (*M *= 8.295, *SD *= 2.299), and BAS-FS (*M *= 4.535, *SD *= 1.591) subscales as covariates, did not reveal a main effect of the emotional condition, *F*(2, 250) = 1.89, *p *= .154, η²_p _= .015. Additionally, none of the impulsivity measures, that is, covariates, showed significant effects on the θ values (all *F *< 1).

Interactions between each impulsivity measure and the emotional condition similarly did not yield significant effects on θ values in any instance (all *F *< 1). As no significant effects were observed, further progress toward post hoc analysis was not pursued.

Table 1 displays the descriptive statistics of the θ values observed in each emotional condition during the time estimation task. It is evident that participants consistently underestimate time across all emotional conditions.

**Table 1.** Mean values (M) and standard deviations (SD) of θ in the three emotional conditions (pleasant, unpleasant, and neutral) of the task of temporal estimation (*n* = 129).

Emotional condition	*M*	*SD*
Pleasant	0.956	0.195
Unpleasant	0.961	0.194
Neutral	0.963	0.202

## Discussion

Impulsivity has been consistently associated with various problematic behaviors and a range of psychopathological disorders, including ADHD and personality disorders. This study aimed to explore the relationship between self-reported impulsivity, as measured by the BIS/BAS, and emotions (pleasant, unpleasant, or neutral) in the context of time estimation deviation. Specifically, the study anticipated: (a) a correlation between higher impulsivity scores, reflected by elevated BAS-FS and BAS-D scores and lower BIS scores, and greater time overestimation; (b) BIS scores being linked to underestimation of time intervals; (c) BAS-FS scores associated with time interval overestimation; (d) BAS-D scores correlating with time interval overestimation; and (e) greater time overestimation in unpleasant emotional conditions compared to pleasant and neutral conditions. Time estimation deviation was evaluated using a time estimation task administered to 129 adult participants recruited from the community, including 88 females.

Although time underestimation was evident across all emotional conditions, analysis using RM ANCOVA did not reveal a main effect of emotional condition, nor were there statistically significant differences observed between the three emotional conditions. Furthermore, none of the impulsivity measures examined in this study, nor their interactions with emotional conditions, showed any effects on time estimation.

Numerous previously published experimental findings concerning the link between impulsivity and time estimation present conflicting results. While a prevalent trend toward time overestimation associated with impulsivity is often reported, individuals with ADHD demonstrate time underestimation across milliseconds to several seconds in various sensorimotor timing tasks (Barkley et al., 2001; Smith et al., 2002). However, they do not differ from healthy individuals in verbal time estimates within the range of several seconds (Burle & Casini, 2001; Kahneman & Tversky, 1979). Notably, these healthy individuals exhibit a greater preference for immediate rewards. Conversely, smokers experiencing strong cravings perceive time as passing more slowly (Sayette et al., 2005), yet they may also exhibit heightened time awareness while awaiting the opportunity to smoke (Sewell et al., 2013). Impulsive individuals with orbitofrontal cortex lesions tend to overestimate time intervals (Berlin et al., 2005; Minkwitz et al., 2012). Similarly, individuals dependent on cocaine or methamphetamine demonstrate time interval overestimation (Wittmann et al., 2007), with this deviation associated with high self-reported impulsivity.

However, there is a scarcity of studies examining how emotions interact with impulsivity and affect individuals’ perception of time passage. It is established that individuals predisposed to boredom, those with depression, or cancer patients experiencing high levels of anxiety perceive time as passing more slowly and tend to overestimate the duration of time estimation tasks (Somov, 2000). Existing literature also suggests that the impact of emotions on TP varies depending on the valence of stimuli. In time estimation tasks, even when controlling for the activation levels of pleasant/unpleasant images, participants generally tend to overestimate the duration of unpleasant images more than that of pleasant ones (Gil & Droit-Volet, 2009; Tipples, 2008). Mechanisms related to attention or induced by activation, proposed to alter TP in impulsive individuals, may not be mutually exclusive but may contribute to altered temporal perception in a complementary manner (Burle & Casini, 2001). Some researchers suggest that the duration of facial expressions of emotion is overestimated compared to neutral expressions, as emotions lead to a state of heightened activation that can accelerate the pace of the internal pacemaker (Dirnberger et al., 2012). These effects might be more pronounced in individuals with higher impulsivity levels, although existing research does not definitively clarify this possibility. It is plausible that our sample lacked individuals with pronounced impulsivity or, at least, did not exhibit variability in impulsivity levels.

If we posit that stable individual differences are grounded in fundamental neuronal processes, the rhythm of an internal timing mechanism emerges as a compelling candidate among the substrates for impulsivity. The perceived acceleration of time passage on a scale of a few seconds may account for the perception of longer time intervals in time estimation tasks, although this effect was not observed in our study. Furthermore, it remains to be determined whether self-reported impulsivity measures predict a preference for immediate rewards over delayed ones, albeit to a lesser extent, and the role of time estimation mechanisms in shaping that preference. Indeed, impulsivity appears to manifest consequences differently across various types of measures, from behavioral assessments conducted in the laboratory to self-report measures derived from questionnaires and scales.

While the obtained results deviate from the existing literature regarding the effects of impulsivity and emotions on TP, several factors could account for these disparities. Firstly, the simplicity of the tasks may have led to a ceiling effect, wherein participants easily reached the maximum performance level. Additionally, the limited number of trials per time slot (15 trials) may have impacted the robustness of the findings. This methodological choice was made in an attempt to minimize participant burden, given the time constraints of the study protocol: 40 min for completing screening instruments (MoCA) and impulsivity assessment (BIS/BAS), in addition to 34 min dedicated to the experimental time estimation task. Furthermore, instrumental constraints also influenced the methodological decisions. Participants responded using a nine-button answer box (keys 1 to 9), thus the task was programed with exposure times ranging from 2 to 7 s (excluding the 8-s option to prevent excessive length), and response options were limited to values between 1 and 9 s. If a response box allowing for more options were available, it is conceivable that the differences between participants would have been more pronounced.

In future investigations, addressing the question of which time intervals are pivotal in TP studies would be paramount. Different interval lengths are likely to exhibit varying degrees of sensitivity to the effects under investigation, contingent upon the specific group of individuals studied and the TP task employed. While this study utilized a time estimation task to assess TP, it would be crucial to incorporate other TP tasks, such as production and temporal reproduction tasks. By examining the effects of impulsivity, psychopathy, and emotional interference across the three most commonly perceived time tasks in the literature (time estimation, time production, and time reproduction), a more comprehensive understanding of temporal perception and its correlates could be attained.

Gaining insight into why individuals with high impulsivity tend to disregard future consequences is a fundamental first step in developing targeted interventions aimed at modifying this behavior, which often leads to adverse outcomes in both daily life and interpersonal relationships. This study has advanced our understanding of how impulsivity interacts with emotions in the context of time estimation. Moving forward, it is crucial to pursue neuropsychophysiological research on impulsive behavior across various stages of human development. Such endeavors will facilitate a more thorough definition and a deeper comprehension of impulsive behavior, thereby shedding light on its complexities (Braquehais et al., 2010; Marusich et al., 2011; Moeller et al., 2001; Morgan et al., 2011).

## Notes

1. The images used were: POSITIVE IMAGES: Faces_104_h, Landscapes_035_v, Objects_074_v, Faces_351_h, Landscapes_064_h, Landscapes_166_h, Faces_116_h, People_169_h, People_154_h, Landscapes_097_v, Landscapes_137_h, Animals_131_h, Faces_140_h, People_185_h, People_043_h; NEGATIVE IMAGES: People_238_h, People_198_h, People_237_h, Faces_371_v, Faces_364_v, People_218_v, Faces_143_v, People_208_h, People_221_h, Faces_365_v, People_220_h, Animals_077_h, People_127_h, Faces_010_h, People_200_h; IMAGENS NEUTRAS: Faces_167_v, Landscapes_170_h, Animals_133_h, Objects_210_h, Animals_081_h, Objects_147_v, Faces_039_h, Faces_216_h, Faces_305_h, Objects_314_h, Objects_057_h, People_146_h, Objects_213_h, Faces_218_h, Objects_112_h.
